# When is it rational to participate in a clinical trial? A game theory approach incorporating trust, regret and guilt

**DOI:** 10.1186/1471-2288-12-85

**Published:** 2012-06-22

**Authors:** Benjamin Djulbegovic, Iztok Hozo

**Affiliations:** 1 Center for Evidence-based Medicine and Health Outcome Research, Clinical Translational Science Institute and Department of Internal Medicine, University of South Florida, Tampa, FL, USA; 2 Departments of Hematology and Health Outcome Behavior, H. Lee Moffitt Cancer Center & Research Institute, Tampa, FL, USA; 3 Department of Mathematics, Indiana University, Gary, IN, 46408, USA; 4 12901 Bruce B. Downs Blvd, MDC02, Tampa, FL, 33612, USA

## Abstract

**Background:**

Randomized controlled trials (RCTs) remain an indispensable form of human experimentation as a vehicle for discovery of new treatments. However, since their inception RCTs have raised ethical concerns. The ethical tension has revolved around “duties to individuals” vs. “societal value” of RCTs. By asking current patients “to sacrifice for the benefit of future patients” we risk subjugating our duties to patients’ best interest to the utilitarian goal for the good of others. This tension creates a key dilemma: when is it rational, from the perspective of the trial patients and researchers (as societal representatives of future patients), to enroll in RCTs?

**Methods:**

We employed the trust version of the prisoner’s dilemma since interaction between the patient and researcher in the setting of a clinical trial is inherently based on trust. We also took into account that the patient may have regretted his/her decision to participate in the trial, while a researcher may feel guilty because he/she abused the patient’s trust.

**Results:**

We found that under typical circumstances of clinical research, most patients can be expected not to trust researchers, and most researchers can be expected to abuse the patients’ trust. The most significant factor determining trust was the success of experimental or standard treatments, respectively. The more that a researcher believes the experimental treatment will be successful, the more incentive the researcher has to abuse trust. The analysis was sensitive to the assumptions about the utilities related to success and failure of therapies that are tested in RCTs. By varying all variables in the Monte Carlo analysis we found that, on average, the researcher can be expected to honor a patient’s trust 41% of the time, while the patient is inclined to trust the researcher 69% of the time. Under assumptions of our model, enrollment into RCTs represents a rational strategy that can meet both patients’ and researchers’ interests simultaneously 19% of the time.

**Conclusions:**

There is an inherent ethical dilemma in the conduct of RCTs. The factors that hamper full co-operation between patients and researchers in the conduct of RCTs can be best addressed by: a) having more reliable estimates on the probabilities that new vs. established treatments will be successful, b) improving transparency in the clinical trial system to ensure fulfillment of “the social contract” between patients and researchers.

## Background

Clinical trials, particularly randomized controlled trials (RCTs), remain an indispensable form of human experimentation as a vehicle for discovery of new treatments [1]. However, since their inception, RCTs have raised ethical concerns [2]. The ethical tension has revolved around consideration of “duty to individuals” vs. “societal value” of RCTs [3,4]. By asking current (trial) patients “to sacrifice for the benefit of future patients” we risk subjugating our duty to consider our patients’ best interest to the utilitarian goal of potentially improving healthcare for the good of others [4,5].

Over the years, equally reasonable, yet vociferous arguments have been made in support of maximizing outcomes for trial patients as opposed to the benefits that future patients will have via conducting testing in adequately performed clinical trials [4]. The debate has, however, crystallized one issue on which all parties agree: in clinical research, particularly research that uses a RCT design, there is interplay between common and conflicting interest of two “players”—a researcher (broadly considered as a representative of society) and a patient. If we accept this premise, then viewed from this perspective, the issue of patients’ enrollment into RCTs (and advances in therapeutics) can be formulated in terms of game theory with two“players” [6]: a patient and a researcher. Game theory has evolved as a branch of applied mathematics as the most suitable technique to model situations that are fraught with conflict and cooperation at the same time [6,7]. It assumes that people act strategically to advance their interests [8]. The best known example of strategic games is the so-called the Prisoner’s Dilemma game (see Section Prisoner’s Dilemma). In its original form, this game is difficult to apply in the context of RCTs[8] because, as discussed below, it does not take trust into account, which is essential for enrollment of patients into experimental clinical trials characterized with hope-for-benefits and unknown harms.

### Box: Prisoner’s Dilemma

Under which circumstances does it pay off to co-operate? The prisoner’s dilemma provides a mathematical solution that addresses the question “when is it more rational to defect vs. cooperate?” The most famous two-person game got its name from the following hypothetical situation: imagine two suspected criminals, Abe and Bill, arrested and isolated for interrogation by the police. Each of them is given an option: (1) testify against their partner in crime (defect) (D); or (2) keep quiet (cooperate with the partner) (C) and ask for a lawyer. Bill does not know what Abe told the police and vice versa.

If only one of the them defects, he gets to walk away as a free man with a $1000 reward, while the other goes to jail for 10 years. If both suspects defect (testify) they will both go to jail for 5 years. On the other hand – if both suspects cooperate with each other (ask for a lawyer) the district attorney has no evidence of a major crime and they both walk away free (but with no reward).

The interesting aspect of this situation is that collectively, both suspects can walk away free if they keep quiet. But strategically, here is what Abe is thinking: (i) if Bill keeps quiet, I better testify in order to get the reward money, (ii) if Bill testifies, I better testify too or I will get 10 years instead of 5. Hence, they both testify and go to jail.

In the medical setting a similar hypothetical situation can be constructed with a busy doctor and her patient. Suppose that a patient comes in with a problem. The doctor has two options: on one hand, he can perform a cursory (5 minute) examination and provide the patient with prescription. Or, the doctor can conduct a thorough exam and give the patient a prescription and management advice after a detailed discussion of benefits and risks of treatments (taking about 60 minutes). The patient can choose to follow the advice and fill the prescription, or to ignore the prescribed treatment and seek a second opinion. There are four possible outcomes:

· (C, C): the doctor spends extra time and gives advice; the patient follows the advice;

· (C, D): the doctor spends extra time and gives advice; the patient seeks a second opinion;

· (D, C): the doctor spends only 5 minutes; the patient fills the prescription;

· (D, D): the doctor spends only 5 minutes; the patient seeks second opinion;

Again, the (C, C) is the best collective outcome. But individually, the patient is better off by seeking a second opinion (as another independent exam is typically best the protection against frequent medical errors), which gives the doctor an incentive to spend as little time on any individual patient as possible and save time. These choices lead to (D,D) as the most logical choice when each “player” decides on their “best” strategy.

When patients enroll in clinical trials they are happy to contribute to knowledge that can help future patients, but, naturally, they also hope to help maximize their own health outcomes [9]. In a similar vein, clinical researchers are primarily motivated to undertake clinical trials to help their own patients. However, these motivations are secondary because the purpose of research is to help future patients [10]. In addition, the history of clinical research is marred by abuses, which indicates that researchers often put their interests ahead of their patients [11-14]. Therefore, enrollment into clinical trials is indeed fraught with common and conflicting interests – those of patients and those of researchers.

The clinical trial, like any other clinical encounter, is fundamentally based on trust [15-17]. In a number of papers, Miller and colleagues argue that there is built-in tension between the goals and interests of researchers and patients who volunteer for clinical trials[14,18-21]. In clinical trials, particularly RCTs, there is an inherent potential for exploiting research participants and abusing trust[14,18-21]; trust is a precondition for human research[16,17].

An important condition for trust is that the truster (i.e., patient) accepts some level of risk or vulnerability [15,22]. If the patient does not believe that the researcher will have her best interest at heart, she will never consent to participate in a clinical trial. Once enrolled in the trial, the patient may discover that her trust was abused [13,23], and will consequently regret that she participated in the trial. Similarly, a researcher may feel guilty because he did not honor the patient’s trust.

In this paper, we employed the trust version of the prisoner’s dilemma game [24] to address the central quandary in clinical research: when is it rational, from the perspective of trial patients and researchers (as societal representatives of future patients), to enroll in clinical trials, particularly in RCTs [4]?

## Methods

### Model

**Figure 1:**
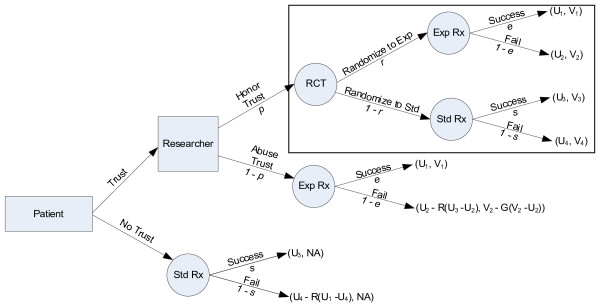
**Model of clinical research according to the trust version of the prisoner’s game dilemma.** The inset shows the equipoise model; e-success of experimental treatment; s-success of standard treatments. R-regret; G-guilt; U1 to U4: the patient’s utilities related to treatment success or failure; V1-V4: the researcher’s utilities related to treatment success or failure; Exp Rx- experimental treatment; Std Rx- standard treatment NA- not applicable (see text for details).

A dilemma whether to enroll in an experimental trial vs. opting for more established treatments can come in the various alternatives. Sometimes, patients themselves may insist on receiving hoped-for but inadequately tested and potentially harmful experimental treatments[25,26]. However, obtaining such treatments would be difficult without the cooperation of a researcher/physician who would need to agree to prescribe such an intervention outside of the clinical trial at the potential risk of professional and personal liability. In addition, these treatments are rigorously controlled by the regulatory agencies such as the Food and Drug Administration [27]. The dilemma can present in the context of participating in phase I, II or III trials. Therefore, one can potentially create many models depending on the specifics of the situation for an individual patient and/or a researcher. We choose to illustrate a dilemma facing investigators and patients by highlighting tension that is commonly encountered in clinical research: should a researcher offer a new, experimental treatment within the context of an RCT, or should he/she offer this promising treatment that is unproven, yet available, outside of the trial [28]? Figure 1 illustrates our model. We believe that the model captures most generic clinical research situations and certainly those that have provoked extensive writings in the medical and ethical literature. [2,29-31]

Although some authors disagree[19,21], most ethicists and clinicians believe that scientific and ethical standards require that a researcher should enroll a patient into a RCT only if there is equipoise, i.e., the honest state of epistemological uncertainty [4,32-39]. When there is such uncertainty, the researchers have ethical and professional obligations to honestly share it with their patients, and as a consequence offer treatment only in the context of RCTs [36-43]. Since no treatment is always successful, we assume that there is a certain probability of success of experimental (*e*) and standard treatment (*s*). The probability of randomization is denoted as *r* in the model. The inset in the Figure 1 represents a model of the classic equipoise model. It should be noted that the ethics of equipoise predominantly focus on the situations when the patient is already considered for enrollment in a RCT: discussion about alternative courses of actions depicted in the rest of Figure 1 rarely occurs [39,44-46]. Similarly, in the equipoise model, the differences in the potential values that patients and researchers may have related to outcomes obtained in a RCT are rarely discussed [39,47]. In fact, it is typically implicitly assumed that researchers’ and patients’ interests are aligned. As a result, and unlike in the proposed trust model (see below), patients and researchers utilities in the equipoise model can be considered equivalent.

However, many researchers strongly believe that one treatment – typically the new one – is superior to the other, and are inclined to offer this treatment directly to their patients, particularly if such a treatment is already available, as for example through the process of the FDA accelerated approval [48,49], or through the regularly approved drugs but for different indications (in so called “off-label” use). Researchers may lean toward offering experimental treatment outside of the trial if they invested considerable effort in helping to develop it. This, however, may lead to direct conflict between the interests of the researcher and his patient [50,51](middle branch in Figure 1). Not surprisingly, researchers’ conflicts of interests – real or perceived– and the well-documented cases of abuse of clinical trial volunteers [13,52], have resulted in alarming publications in the lay press about the use of humans as “guinea pigs” solely for the purpose of advancing science and scientific careers [53]. However, the nature of experimental studies is such that the patients cannot be guaranteed successful outcome with any treatment [4,32]. Hence, as outlined in the Introduction, clinical trials, like any other clinical encounter, are inherently based on trust [15]. Analogous to the lack of guaranteed outcomes, patients cannot be guaranteed in advance that their trust will not be abused. The probability that trust will be honored is denoted as “*p*” in the model. Hence, if a patient does not believe that her best interests are not the primary focus of her physician-investigator, she will never consent to participation in a clinical trial. As a consequence, the patient will request established, standard treatment (the lower branch in the model). (NB the model does not distinguish between a researcher’s honest beliefs that “his” experimental treatment is superior to standard therapy from his conscious or subconscious bias in favor of experimental therapy. The model does, however, implicitly assume that if *p = 0*, the researcher always believes that the experimental treatment is better than standard treatment).

However, after the patient is enrolled in the trial, she may discover that her trust was abused. Consequently, the patient will regret having volunteered participation in the trial. Regret (R) is defined as a fraction of the difference between the utility of the best action taken and the utility of the outcome we should have taken, in retrospect [54-56]. In this model, we expressed regret as a fraction of the loss of potential utilities [24]. Although regret under the scenarios of “Do Not Trust” and “Abuse Trust” is likely different, we kept the scenarios identical in our model for simplification purposes. Similarly, a researcher may feel guilty (*G*) because he abused the patient’s trust. Guilt expresses the psychological reaction of a researcher abusing the trust of the patient. The guilt diminishes the researcher’s utility by a fraction of the difference between the researcher’s and the patient’s utilities under the “Honor Trust” vs. “Abuse Trust” scenarios” in Figure 1[24].

Each of the alternative courses of action shown in Figure 1 is associated with the payoffs (utilities). The utilities are likely different between the patients (*U*) and researchers (*V*). If the utilities are the same, then the prisoner’s dilemma model does not apply. In fact, in the case of the scenario shown in the inset, the tree reduces to the equipoise model discussed above. In our trust model we assumed that *U*_1_ ≥ *U*_3_ ≥ *U*_2_, *U*_4_ and *V*_1_ ≥ *V*_2_, *V*_3_ ≥ *V*_4_. We assumed that utilities associated with treatment success must be higher than those related to treatment failure. We also assume that *V*_2_ ≥ *U*_2_ since society benefits from knowledge obtained even in cases of unsuccessful testing (e.g., such knowledge helps to avoid administration of the unsuccessful treatments to future patients, and to allocate resources to the development of other therapies that look more promising, etc.). Finally, we assumed that the “game” can be played only once, i.e., the same patient will not be enrolled in more than one trial. Although some patients can indeed be invited to participate in more than one trial, in contemporary clinical research practice, the vast majority of patients are enrolled in one trial only.

### Data

A few published studies have addressed the question of the probability of success of experimental vs. established, standard treatments [57]. In the largest study to date, which synthesized data from RCTs performed over 50 years in the field of cancer, we found that the overall probability of success of experimental vs. standard treatments was 41% vs. 59%, respectively [58]. Similar results were reported in other fields[59]. These data support the theoretical requirement for equipoise before offering enrollment into RCTs, and indicate that, regardless of the field, disease or a type of interventions, the probability of success of new vs. established treatment should be about 50:50, which is what we assumed in the equipoise model (see the inset, Figure 1)[4,32,60,61] Empirical studies showed that fewer than 3% of patients would accept randomization if the probability of success of one treatment over another exceeded 80% [62,63]. Hence, we varied the values describing treatment success in our trust model (variables *e* and *s*, respectively) over the 20-80% range (Table 1). The model assumes that both patients and researchers are more interested in the therapeutic “success” of treatment (i.e., whether experimental treatment is “better” than standard treatment and vice versa) than in knowing the precise magnitude of treatment itself in terms of hazard ratio, relative risk, etc.

**Table 1:** Data

**Variable**^**#**^	**Researcher**	**Patient**	**Trial**
**Utilities**^$^			
Success of experimental treatment within trial*	95 (80–100)	90 (50–100)	
	Assumed 100(1) in the equipoise model (inset)	Assumed 100(1) in the equipoise model (inset)	
	(MC modeling: triangular distribution)	(MC modeling: triangular distribution)	
Failure of experimental treatment within trial*	54 (10–100)	16.3 (0–50)	
	Assumed 0 in the equipoise model (inset)	Assumed 0 in the equipoise model (inset)	
	(MC modeling: triangular distribution)	(MC modeling: triangular distribution)	
Success of standard treatment within trial*	70 (40–80)	84 (50–1100)	
	Assumed 100 (1) in the equipoise model (inset)	Assumed 100 (1) in the equipoise model (inset)	
	(MC modeling: triangular distribution)	(MC modeling: triangular distribution)	
Failure of Standard treatment within trial*	44 (0–80)	16.9 (0–50)	
	Assumed 0 in the equipoise model (inset)	Assumed 0 in the equipoise model (inset)	
	(MC modeling: triangular distribution)	(MC modeling: triangular distribution)	
**Probabilities**			
Success of experimental treatment (e)			0.41 (0.2-0.8)**
			(0.5 for the equipoise model)
			(MC modeling: binomial distribution; n = 450***)
Failure of experimental treatment			1-e
Success of standard treatment (s)			0.59 (0.2-0.8)**	
			(MC modeling: binomial distribution; n = 316***)	
Failure of standard treatment			1-s	
Randomization (r)			0.5 (0.2-0.8)	
			(0.5 for the equipoise model)	
			(MC modeling: triangular distribution)	
Honoring trust (p)			0.5 (0–1)	
**Regret**		0.2 (0–1)		
		(MC modeling: triangular distribution)		
**Guilt**	0.2 (0–1)			
	(MC modeling: triangular distribution)			

^# -^ sensitivity analysis was performed for the values shown in Table but also for all extreme values (0–1)- the change in the assumptions did not affect the results of the analysis; ^$^- based on the survey of 8 clinical investigators; * assumed to be the same within and outside of the trial; MC- Monte Carlo; **- actual values based on reference [41] is 0.41 (0.37-0.45) for experimental and 0.59 (0.55-0.62) for standard treatment, respectively ***-based on the reference [41].

No empirical data exist that can precisely inform the values of each of the utilities in our model. Therefore, we surveyed a convenience sample of 8 experienced clinical investigators, asking them to provide the values for each of utilities shown in Figure 1, first from a patient and then from a researcher perspective. Since the values did not substantially differ for the utilities of treatment success and failure within and outside of the clinical trial, respectively, we further simplified the model by using the same values for utilities for each of these scenarios shown in Figure 1. Table 1 shows the summary of the utilities based on this survey with a wide range of the values in sensitivity analysis. By making the ranges wide, the lack of empirical data becomes a less important issue since the results informed by putative empirical information would most likely fit within the range of our analysis (see Results).

### Analysis

We first identified ranges for the variables for which the ***best strategy*** for the patient is to trust or not to trust a researcher, and the researcher to have incentive to honor or abuse the patient’s trust. We complemented this analysis by employing the Monte-Carlo modeling technique, varying all variables over the values shown in Table 1. We ran the analysis for 100,000 trials. The latter analyses were performed using the Microsoft EXCEL software.

## Results

1. The equipoise modelAssuming that the utilities and probabilities between experimental and standard treatments are equal, as theoretically predicted[4,32] (Table 1), the solution of the tree shown in the inset of Figure 1 provides the following simple equation:

(1) $$r=\frac{1}{1+\frac{e}{s}}=0.5$$

The equipoise model, thus, indicates that the most rational solution for a researcher to offer and for a patient to accept randomization occurs at the probability of 50%.

2. The trust modelWe solved the entire tree shown in Figure 1 both from the researchers’ and patient’s point of view.

a) From the researcher’s point of view:The expected value of “Honor Trust” and enroll the patient in a RCT is given as :

(2) $$\begin{array}{l} E\left[Honor\right]=rE_{V}\left[Exp\right]+\left(1-r\right)E_{V}\left[Std\right]\\ E\left[Abuse\right]=eV_{1}+\left(1-e\right)\left(V_{2}-G\cdot \left|V_{2}-U_{2}\right|\right)=E_{V}\left[Exp\right]-\left(1-e\right)G\cdot \left(V_{2}-U_{2}\right) \end{array}$$

where *E*_*V*_ [*Exp*] *and E*_*V*_ [*Std*] are the expected value, to the researcher, of the experimental and standard treatment, respectively.Therefore, the expected value of “Honor Trust” is larger than the expected value of “Abuse Trust” if:

(3) $$\begin{array}{l} E\left[Honor\right]\geq E\left[Abuse\right]\\ rE_{V}\left[Exp\right]+\left(1-r\right)E_{V}\left[Std\right]\geq E_{V}\left[Exp\right]-\left(1-e\right)G\cdot \left(V_{2}-U_{2}\right)\\ r\left(E_{V}\left[Exp\right]-E_{V}\left[Std\right]\right)\geq E_{V}\left[Exp\right]-E_{V}\left[Std\right]-\left(1-e\right)G\cdot \left(V_{2}-U_{2}\right) \end{array}$$

If *E*_*V*_ [*Exp*] ≥ *E*_*V*_ [*Std*], the inequality above means that the researcher will have a higher expected value of honoring trust if

(4) $$r\geq 1-\frac{\left(1-e\right)G\cdot \left(V_{2}-U_{2}\right)}{E_{V}\left[Exp\right]-E_{V}\left[Std\right]}$$

On the other hand, if *E*_*V*_ [*Exp*] < *E*_*V*_ [*Std*], the inequality above means that the researcher will have higher expected value of honoring trust if

(5) $$r<1-\frac{\left(1-e\right)G\cdot \left(V_{2}-U_{2}\right)}{E_{V}\left[Exp\right]-E_{V}\left[Std\right]}$$

b) From the patient’s point of viewThe expected value of “Do Not Trust” is straightforward:

(6) $$E\left[No\;Trust\right]=sU_{3}+\left(1-s\right)\left(U_{4}-R\left(U_{1}-U_{4}\right)\right)=E_{U}\left[Std\right]-\left(1-s\right)R\left(U_{1}-U_{4}\right)$$

The expected value of trust is equal to

(7) $$\begin{array}{l} E\left[Trust|Honor\right]=rE_{U}\left[Exp\right]+\left(1-r\right)E_{U}\left[Std\right]\\ E\left[Trust|Abuse\right]=eU_{1}+\left(1-e\right)\left(U_{2}-R\left(U_{3}-U_{2}\right)\right)=E_{U}\left[Exp\right]-\left(1-e\right)R\left(U_{3}-U_{2}\right) \end{array}$$

where *E*_*U*_ [*Exp*] and *E*_*U*_ [*Std*] are the expected value of the experimental and standard treatment, respectively, for a patient.If *p* is the percentage of times (or estimated subjective probability) that the researcher honors trust (i.e. to act in the patient’s best interest by offering her enrollment into an RCT), the expected value of “Trust” is:

(8) $$\begin{array}{l} E\left[Trust\right]=p\left(rE_{U}\left[Exp\right]+\left(1-r\right)E_{U}\left[Std\right]\right)+\left(1-p\right)\left(E_{U}\left[Exp\right]-\left(1-e\right)R\left(U_{3}-U_{2}\right)\right)\\ =prE_{U}\left[Exp\right]+p\left(1-r\right)E_{U}\left[Std\right]+\left(1-p\right)E_{U}\left[Exp\right]-\left(1-p\right)\left(1-e\right)R\left(U_{3}-U_{2}\right)\\ =E_{U}\left[Exp\right]+p\left(1-r\right)\left(E_{U}\left[Std\right]-E_{U}\left[Exp\right]\right)-\left(1-p\right)\left(1-e\right)R\left(U_{3}-U_{2}\right)\\ =p\left(1-r\right)\left(E_{U}\left[Std\right]-E_{U}\left[Exp\right]\right)+p\left(1-e\right)R\left(U_{3}-U_{2}\right)+E_{U}\left[Exp\right]-\left(1-e\right)R\left(U_{3}-U_{2}\right)\\ =p\left(\left(1-r\right)\left(E_{U}\left[Std\right]-E_{U}\left[Exp\right]\right)+\left(1-e\right)R\left(U_{3}-U_{2}\right)\right)+E_{U}\left[Exp\right]-\left(1-e\right)R\left(U_{3}-U_{2}\right) \end{array}$$

Therefore, *E*[*Trust*] ≥ *E*[*No Trust*] if

(9) $$\begin{array}{c} p\left(\left(1-r\right)\left(E_{U}\left[Std\right]-E_{U}\left[Exp\right]\right)+\left(1-e\right)R\left(U_{3}-U_{2}\right)\right)+E_{U}\left[Exp\right]-\left(1-e\right)R\left(U_{3}-U_{2}\right)\\ \geq E_{U}\left[Std\right]-\left(1-s\right)R\left(U_{1}-U_{4}\right) \end{array}$$

**Figure 2:**
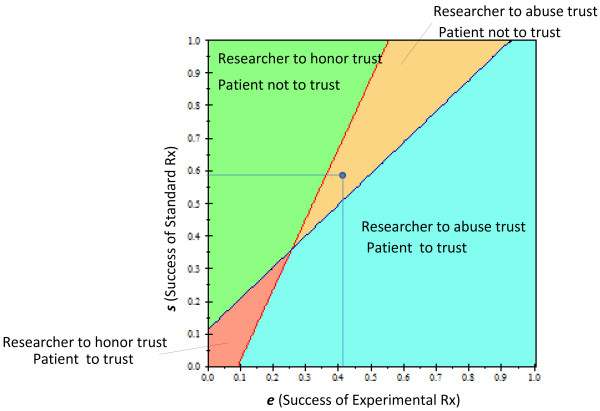
**Two-way sensitivity analysis of the prisoner’s dilemma trust game of clinical trials.** The effect of the probability of treatment success on: **a**) the patient’s trust of the researcher (whether to enroll in the trial), **b**) researcher’s inclination to honor the trust. At the intersection, the two strategies are identical. The dot shows the baseline values of the model. Color fields indicate the optimal strategy for each player.

**Figure 3:**
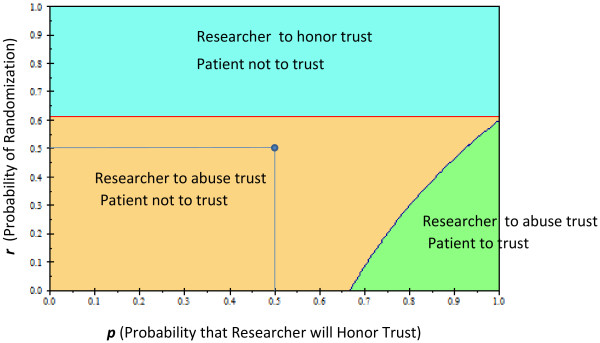
**Two-way sensitivity analysis of the prisoner’s dilemma trust game of clinical trials.** The effect of the probability of randomization to a particular treatment and the probability that the researcher will honor the trust on: **a**) patient’s trust whether to enroll in the trial, **b**) the researcher’s inclination to honor the trust. The dot shows the baseline values of the model. Color fields indicate the optimal strategy for each player.

The solution of the tree under the baseline values shown in Table 1 produced a disconcerting result: the most rational behavior for the patient is to not trust and to not enroll in the RCT [Expected value (EV_trust_) = 45 vs. EV_no_trust_ = 50]. From the researcher’s point of view, the strategy with the highest expected utility was associated with “Abuse Trust” Expected value(EV_honor_trust_) = 65 vs. EV_abuse_trust_ = 66].The analysis was sensitive to most assumptions about the utilities related to the success and failure of therapies that are tested in RCTs. Figure 2 displays the results of the patient’s and researcher’s strategy over all possible values of the success of experimental and standard treatments. Under the baseline assumption of the model, the most rational strategy for the patient is not to trust and for the researcher to abuse this trust. It can also be seen that the higher the probability that the experimental treatment will be successful, the more incentive the researcher has to abuse the patient’s trust. However, for the wide range of success of experimental treatments, the most rational strategy is still for the patient to trust, despite the possibility that the researcher may not honor it: the likelihood of obtaining successful treatment appears to justify putting oneself in a vulnerable position. Figure 3 shows the two-way sensitivity analysis for *p* (probability that the researcher will honor trust) vs. *r* (probability of randomization). Under typical randomization of 50%, in the trust model, unlike in the equipoise model, the most rational strategy for the researcher and patient is not to cooperate. The researcher has incentive to honor trust only when the probability that a patient’s chance of being randomized to the experimental treatment is ≥61%. On the other hand, the patient should rationally exercise his/her trust only if the probability that the researcher will honor the trust is ≥67%. We also observed two other interesting results: under our assumption that regret is not greater than guilt, neither the patient’s regret nor the researcher’s potential guilt affected the analysis. In addition, the results were not affected by the patient’s utility related to the success of experimental treatment (it would have to be >100% in order to override any patient’s concern about trustworthiness of the researcher) (Results not shown).

3. Monte Carlo AnalysisBy varying all variables in the Monte Carlo analysis we found that patients are inclined to trust researchers, and that researchers honor that trust in only 19% of trials (Table 2). That is, under the assumptions of our model, enrollment into an RCT represents a rational strategy that can meet both patients’ and researchers’ interests simultaneously 19% of the time. On average, the researcher can be expected to honor trust 41% of the time, while the patient is inclined to trust the researcher 69% of the time.

**Table 2:** Trust Game: Results of Monte-Carlo Analysis

	**Trials**	**Patient**		
	**(100,000)**			
		Trust	No Trust	TOTAL
		18867	21911	40778
	Honor	(18.9%)	(21.9%)	(40.8%)
**Research**		50014	9208	59222
	Abuse	(50.0%)	(9.2%)	(50.2%)
		68881	31119	100,000
TOTAL		(68.9%)	(31.1%)	(100%)

## Discussion

In this paper, we used the game theory approach to model the clinical trial encounter. Given that the clinical trial interaction, as with any other medical interaction, is inherently based on trust, i.e., represents a *bona fide* relation between a patient and a researcher, we employed the trust version of the prisoner’s game dilemma [24]. This approach is based on the “risk-assessment views” of trust, in which trusting is rational under certain conditions that are expected not to lead to the betrayal of our trust [15,22]. This view stresses the importance of having reliable evidence about conditions in which we find ourselves when we deliberate about whether to accept some level of risk or vulnerability when we place our well-being in the hands of others [15,22]. Trust is an epistemic cause – we cannot simply want to trust without an evidentiary basis justifying it [15,22].

Under the baseline conditions of our model, our analysis generated an unsettling finding: both patient’s and researcher’s expected utility value was the highest for the scenario “Do not trust” and “Abuse trust”, respectively. This finding holds out despite the fact that the difference between two strategies in terms of the numerical results was rather minimal (see Results). This is because from a decision-analytic point of view, we should choose the strategy most likely to give us the best outcome, regardless of whether we believe it will be superior 51% or 99% of the time[64]. Thus, from the *individual* point of view, in trying to decide whether to enroll in a *single* trial, the most rational behavior is not to cooperate. It is possible that this type of behavior can explain the low rate of enrollment into clinical trials. For example, fewer than 3% of patients eligible for participation in clinical trials enroll in them [65].

We were surprised to find out that over the wide range of assumptions, the probability of randomization plays a relatively smaller role than anticipated and becomes important only when it exceeds 61% (Figure 1). This is in contrast to the equipoise model (Figure 1, inset) where 50% of randomization represents the right value, helping reconcile the theory of human experimentation with the theory of rational choice [4]. These findings indicating that randomization itself may be less ethically important than previously recognized are interesting in light of vociferous debate about the role of randomization in human experimentation [2,29,30,44,66]. The reason for the shift can be best understood by inspecting Figure 1: the key decision related to participation in research begins with the assessment of how effective current “standard” treatments are [31]. Depending on the assessment of information related to the effects of established treatments, the patient will decide whether to consider enrollment into a clinical trial. Therefore, having reliable evidence on the benefits and risks of the currently available treatments becomes critical not only for the practice of medicine but for participation in clinical trials – the importance of this knowledge has long been stressed by the proponents of the evidence-based medicine movement [40,41,67,68]. Indeed, the assessment of the probability of success of experimental vs. standard treatment proved to be a much more important variable in our model, both for the patient and the researcher (Figure 3). In 1995, Chalmers called for reliable estimates of the probabilities of treatment success as the key ethical requirement for enrollment into RCTs [57]. To date, a few systematic analyses have been performed on this important topic. In the largest study to date [58], we estimated the probability of success of new, innovative vs. established treatments, which was somewhat dependent on the type of metrics used. Using a meta-analytic technique, we estimated that the probabilities of success of experimental vs. standard treatments are about equal, 50%:50%. In the analysis reported in this paper, we employed the 41% vs. 59% figure, which was based on the global researchers’ assessment of the superiority of treatments [58] (See Table 1). Under assumption of the baseline success rate of 59%:41%, the most rational behavior is not to cooperate. Under the assumption of a 50%:50% success rate, the patient’s rational behavior is to trust the researcher, while the researcher has incentive to dishonor the patient’s trust (Figure 2). It is interesting that we can expect full cooperation only in situations where the expected success rates of experimental and standard treatment are low (Figure 2). In all other situations, conditions are created for either the patient not to trust or the researcher to abuse the trust.

A number of historical abuses of patients who volunteered as research participants has contributed to the erosion of trust in medical profession [13,23], and they provide empirical justification for our model. The situation can be improved by cultivating trust and enforcing the social contract view of trust[15-17,69]. This should be the goal of oversight policies, including the requirement for mandatory training in human subject research, reducing conflicts of interest, etc. One way to minimize potential abuses on the part of researchers is to enforce norms of expected behavior[15,70]. These policies should be coupled with more transparency in human clinical research, and with obtaining better evidence on the actual benefit and risks of participating in research. These measures would go a long way to boosting patients’ trust in the system and ultimately lead to higher levels of participation in clinical research. The goal would be to align patients’ and researchers’ interests. This alignment would ultimately create conditions that promote the spirit of co-operation, in which participation in clinical investigations is viewed as a critical way to support this important public good. These conditions should also support the idea that we all have a duty to participate in research, unless there is a good reason not to [71].

Our model has some limitations. First, we considered only one type of clinical scenario. As explained above, it is possible to model many other scenarios. Nevertheless, we believe we chose the most typical clinical research situation, making our model relevant to most ethicists and clinical scientists. Second, we lack empirical data on most of the variables used in the model, particularly on the patients’ utilities. However, we used a wide range for the values in our analysis, which almost certainly would include such putative empirical data should they be obtained. We also think this type of research would be very difficult to subject to empirical testing, and modeling is the probably the best approach we will ever have to tackle the important ethical issue presented in this paper. This is particularly true since most researchers would have difficulty admitting guilt associated with the abuse of the patient’s trust. Similarly, it would be difficult to measure the patient’s regret, although our model indicates the lack of its importance. It is still, however, possible that focusing on regret associated with the process [72,73] rather than outcomes, as we did, could make the role of regret more important than our results indicate. Third, we employed a relatively narrow view of the trust-risk assessment model. Thus, our model lacks a broader societal perspective and integration of other important elements of trust such as virtue, goodwill or moral integrity[15]. We think this is important, but building such a model would be immensely more complicated, and is beyond of the scope of this paper.

## Conclusions

In conclusion, we found that under the majority of typical circumstances in clinical research today, most patients can be expected not to trust researchers, and most researchers can be expected to abuse the patients’ trust. The situation can be improved by: a) having more reliable estimates of the probability that a new treatment will be more successful than an established treatment, b) improving transparency in the clinical trial system, c) enforcing “the social contract”[69] between patients and researchers by minimizing conflicts of interest, maintaining oversight and mandating continuing training in human subject research. These efforts will likely lead to decreases in the well-documented abuses in clinical research while improving participation in clinical trials.

## Competing interests

'The author(s) declare that they have no competing interests'.

## Authors’ contribution

BD had an idea for the study. BD & IH jointly developed the model. IH solved the model and performed the analyses. BD wrote the first draft. Both authors read and approved the final manuscript.

## Pre-publication history

The pre-publication history for this paper can be accessed here:

http://www.biomedcentral.com/1471-2288/12/85/prepub
